# Chemoradiation-induced alteration of programmed death-ligand 1, CD8+ tumor-infiltrating lymphocytes and mucin expression in rectal cancer

**DOI:** 10.18632/oncotarget.28255

**Published:** 2022-07-28

**Authors:** Marina Baretti, Qingfeng Zhu, Wei Fu, Jeffrey Meyer, Hao Wang, Robert A. Anders, Nilofer S. Azad

**Affiliations:** ^1^Department of Oncology, Sidney Kimmel Comprehensive Cancer Center, Johns Hopkins University School of Medicine, Baltimore, MD 21287, USA; ^2^Department of Pathology, Johns Hopkins University School of Medicine, Baltimore, MD 21287, USA; ^3^Division of Biostatistics and Bioinformatics, Sidney Kimmel Comprehensive Cancer Center, Johns Hopkins University School of Medicine, Baltimore, MD 21287, USA; ^4^Department of Radiation Oncology and Molecular Radiation Sciences, Johns Hopkins University School of Medicine, Baltimore, MD 21287, USA

**Keywords:** programmed death ligand 1, tumor-infiltrating lymphocytes, immune checkpoints, colorectal cancer, neoadjuvant chemoradiotherapy

## Abstract

Introduction: DNA damage and resulting neoantigen formation is considered a mechanism for synergy between radiotherapy and PD-1/PD-L1 pathway inhibition to induce antitumor immune response. We investigated neoadjuvant chemoradiotherapy (nCRT)-induced changes in CD8+ tumor infiltrating lymphocyte, PD-L1 and mucin expression in rectal cancer patients.

Materials and Methods: Tumor samples of rectal adenocarcinoma patients undergoing resection between 2008-2014 with (*n* = 62) or without (*n* = 17) nCRT treatment were collected. Sections were stained with CD8 and PD-L1 antibodies for immunohistochemistry. The prevalence of CD8+ cells was recorded in the tumor, interface tumor and background rectal side. Image analysis was used to determine the density of CD8+ lymphocytes. The percentage of PD-L1 expression was manually counted in tumor cells (TC), tumor stroma (TS) and the invasive front (IF). Mucin expression was determined as the percentage of the mucin area in the whole tumor area.

Results: PD-L1 expression on TCs was identified in 7.6% (6/79) of nCRT specimens (*p* = 0.33) and in none of the non-nCRT patients. Median densities of CD8+ infiltrating T lymphocytes did not differ significantly between the two groups. Mucin expression was significantly higher in the nCRT cohort (*p* = 0.02). Higher neutrophil to lymphocytes ratio (NLR) after nCRT was associated with worse outcome (HR = 1.04, 95% CI = 1.00–1.08).

Conclusions: nCRT exposure was associated with a non-significant difference in PD-L1 expression in rectal adenocarcinoma patients, possibly due to sample size limitations. Further mechanistic investigations and comprehensive immune analysis are needed to understand nCRT-induced immunologic shift in rectal cancer and to expand the applicability of checkpoint inhibitors in this setting.

## INTRODUCTION

Colorectal cancer (CRC) is the second most common cause of cancer death in the United States and rectal cancer comprise 44% of CRC [1]. Standard treatment for locally advanced rectal cancer (LARC) consists in neoadjuvant (preoperative) chemoradiotherapy (nCRT) combined with surgery [2], with the aim to reduce local recurrence and to increase the sphincter preservation rate. However, only a minority of patients achieve a complete response after nCRT treatment [3].

Antitumor activity of Radiation therapy (RT), and chemoradiotherapy (CRT), is thought to be partly due to the activation of tumor-specific adaptive immunity [4, 5]. Upon radiation, there is upregulation of up- major histocompatibility complex molecules [6, 7] and release of tumor associated antigens [8] which in turns leads to release of inflammatory cytokines, especially interferon-γ (IFN-γ) from tumor and immune cells [9]. As results, the immunologic equilibrium of the tumor microenvironment (TME) is shifted towards a more immunogenic one [10, 11]. However, activation of immune suppressive pathways, including PD-1/PD-L1 pathway also happens, limiting the potential beneficial effects of this increased immunogenicity [12–16].

Recent preclinical studies showed that combining PD-1/PD-L1 inhibitors with CRT improved both local and systemic tumor control in animal models [17–20]. The synergistic effects of CRT and PD-1/PD-L1 immunotherapy has been supported by several retrospective analyses in different cancer types, including esophageal cancer, bladder cancer, and lung cancer also support [14, 21, 22]. However, the role of nCRT to interact synergistically with for immune checkpoint inhibitor treatment to improve tumor control in rectal cancer remain uncertain. Additionally, controversies exist regarding the prognostic value of PD-L1 expression in rectal cancer. The aim of this study was to evaluate nCRT-induced alterations in the TME of post-CRT resected specimens of rectal cancer, with a particular focus on PD-L1 expression and the density of CD8+ tumor-infiltrating lymphocytes (TILs). We used rectal cancer cases where nCRT was not delivered as control cases. We also examined the densities of CD8+TILs and PD-L1 expression on the basis of their localization. Finally, we aimed to analyze mucin expression within the tumor, as mucins might play an important role in inflammation and immune responses.

## RESULTS

### Clinicopathologic characteristics

From 2008 and 2014, 79 patients with rectal adenocarcinoma were included in this analysis who were treated with surgery with or without nCRT at the Johns Hopkins Hospital and had resection specimens available for analysis. Baseline patient and tumor-related characteristics of the study group are given in Table 1. The median age was 52 years (range, 21–88 years), with majority of male patients (70%) were men. Most patients (58%) had pT3 tumors. At time of diagnosis 50 patients (63%) had node-positive disease and 41 patients (52%) had pathologically positive nodal status.

**Table 1:** Clinicopathologic characteristics

Characteristic	*n* (%)
Age (y)	
Median (range)	52 (21–88)
<52	38 (48)
≥52	41 (52)
Sex	
Male	55 (70)
Female	24 (30)
Tumor location	
Rectum	62 (78)
Recto-sigmoid	17 (21)
Unknown	1 (1)
Clinical Stage at Diagnosis	
Stage 1	7 (9)
Stage 2	9 (11)
Stage 3	50 (63)
Stage 4	13 (17)
Tumor grade (resected)	
Well to moderately differentiated	60 (76)
Poorly- differentiated	9 (11)
NE (no residual tumor)	10 (13)
pT stage	
T0	3 (4)
T1	3 (4)
T2	17 (21)
T3	46 (58)
T4	7 (9)
NA	3 (4)
pN Stage	
Nx	3 (4)
N0	35 (44)
N1	25 (32)
N2	16 (20)
N3	0 (0)
Downstage of after CRT, *n* = 61	
Yes	25 (41)
No	31 (51)
NE	5 (8)
Lymphatic Invasion	
Yes	24 (30)
No	32 (40)
Unknown	18 (23)
Vascular Invasion	
Yes	7 (9)
No	57 (72)
Unknown	15 (19)
Microsatellite instability	
MSS/MSI-Low	42 (53)
MSI-H	1 (1)
Unknown	36 (46)

Values are number (percentage) unless otherwise noted. Abbreviations: CRT: chemoradiation; MSI-H: microsatellite instability-high; MSS: microsatellite stable; NE: not evaluable.

Significant differences were found between the two groups in terms of clinical stage at the time of diagnosis: patients in the nCRT arm had more advanced stage at the time of diagnosis, with 50 patients (92.6%) diagnosed with stage 3 or 4 disease and 4 (4,2%) with stage 1 or 2 cancer. In the groups that did not receive nCRT (*N* = 17), 7 patients had stage 3 or 4 disease, while 10 (58.8%) were diagnosed with stage 1 or 2 cancer (*p* = 0) (Table 2). Other clinical features were assessed for imbalance, and arms were well matched.

**Table 2:** Stage at the time of diagnosis, PD-L1, CD8+TILs and mucin expression by radiation therapy

	NO nCRT	nCRT	*p*-Value
**Stage at Diagnosis, *n* (%)**	*N* = 17	*N* = 54	**0**
1, 2	10 (58.8)	4 (7.4)	
3,4	7 (41.2)	50 (92.6)	
**PD-L1 TC, *n* (%)**	*N* = 17	*N* = 61	0.329
neg	17 (100)	55 (90.2)	
pos	0 (0)	6 (9.8)	
**PD-L1 IF**	*N* = 17	*N* = 61	0.942
median	25	10	
mean	30.9	30.2	
**PD-L1 TS, *n* (%)**	*N* = 17	*N* = 61	0.559
Neg	6 (35.3)	17 (27.9)	
Pos	11 (64.7)	44 (72.1)	
**PD-L1 Interface, *n* (%)**	*N* = 17	*N* = 61	0.558
Neg	4 (23.5)	21 (34.4)	
Pos	13 (76.5)	40 (65.6)	
**CD8+ Intratumor,**	*N* = 15	*N* = 49	0.793
Median	105.8	99.5	
Mean	229.3	207.1	
**CD8 inner interface, *N***	*N* = 9	*N* = 29	0.47
Median	131	173.5	
Mean	348.8	220.9	
**CD8 outer interface, *N***	*N* = 7	*N* = 28	0.2156
Median	330	202	
Mean	634	283.3	
**Mucin ratio**	*N* = 2	*N* = 9	**0.02**
Median	0	0.3	
Mean	0	0.2	

Abbreviations: nCRT: neoadjuvant chemoradiation; IF: interface; TC: tumor cells; TS: tumor stroma.

nCRT consisted of fluoropyrimidines- based chemotherapy administered concomitant with RT. Radiotherapy consisted of 50.4 Gy radiation in 28 fractions delivered to the primary tumor and the mesorectal, pre-sacral and internal iliac lymph nodes. The median time interval between n CRT and surgery (total mesorectal excision) was 63.3 days. Pathologic downstaging was observed in 25 patients (41%). Mismatch repair status was available for 43 patients (53%) and only one patient was found to have microsatellite unstable diseases (1%).

### PD-L1, CD8+ TILs and mucin expression with and without neoadjuvant radiochemotherapy

**Figure 1:**
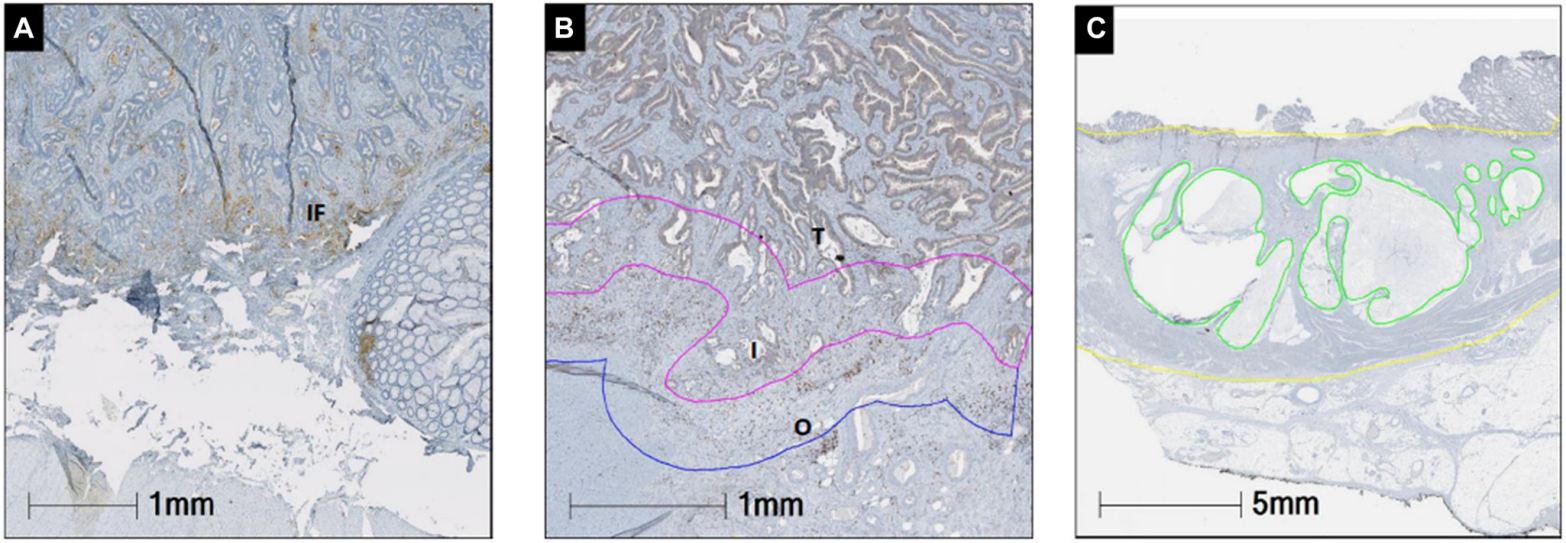
Representative images of PD-L1 (**A**), CD8 (**B**) and mucin (**C**) and staining. The percentage of PD-L1 membranous expression was manually counted in tumor cells tumor stroma and invasive front. The prevalence of CD8+ TILs was recorded in tumor, interface tumor side (inner), interface background rectal side (outer). Mucin expression (green) was determined as percentage of the mucin area in the whole tumor area mass (yellow). Abbreviations: I: inner; IF: invasive front; O: outer front; T: tumor.

PD-L1 expression in rectal adenocarcinoma cells and immune cells was evaluated in the surgical specimens of the two patient cohorts: patients who did receive nCRT before surgery (*n* = 61) and patients who had surgery without nCRT (*n* = 17). One patient received only chemotherapy and not radiation before surgery and was excluded from analysis. PD-L1 expression was studied according to spatial localization: tumor cells (TC), tumor stroma (TS) and invasive front (IF). Figure 1A–1C shows the representative slide views. Considering the overall population, both the maximum staining intensity and the proportion of PD-L1-expressing cells were more pronounced in the TS (70.5%) and IF (67.9%).

PD-L1 expression on TCs was identified in only 7.7 % (6/79) of specimens. All 6 cases had received nCRT (*p* = 0.33) (Table 2). Generalized estimating equation (GEE) was used to account for the repeated measures obtained from the same patient. The results of GEE are consistent with what we found using Chi-square test, except for PD-L1: comparisons between the patient groups with and without nCRT showed a significant difference in PD-L1+ rectal cancer cells (*p* = 0) (Table 3).

**Table 3:** PD-L1 expression by radiation therapy using GEE (generalized estimating equations) analysis

	NO nCRT (*N* = 17)	nCRT (*N* = 61)	*p*-Value
**PD-L1 TC, *n* (%)**			0*
Neg	17 (100)	55 (90.2)	
Pos	0 (0)	6 (9.8)	
**PD-L1 IF**			0.94
Median	25	10	
Mean	30.9	30.2	
**PD-L1 TS, *n* (%)**			0.56
NEG	6 (35.3)	17 (27.9)	
Pos	11 (64.7)	44 (72.1)	

Abbreviations: nCRT: neoadjuvant chemoradiation; IF: interface; TC: tumor cells; TS: tumor stroma.

CD8+ tumor-infiltrating T cells, were evaluated by IHC staining within the tumor and the interface (inner and outer interface). No significant differences in CD8+T cells density by location (tumor or interface) were observed in the two cohorts of patients (Table 2).

Differences in CD8+T cells infiltrated between surgery-alone and nCRT cases were observed, although not statistically significant: the median density (cells/mm^2^) of CD8+ TILs was 319.66 (range, 20.76–978.08) in the surgery alone group and 787.05 (range, 101.39–2100.85) in the nCRT, respectively.

Mucin ratio, defined as mucin area versus total tumor area (Figure 1C), was evaluable in a total of 11 specimens, and was significantly higher in the cohort of patient who received nCRT (median 0.3 versus 0, *p* = 0.02) (Table 2).

### Independent prognostic risk factors

Among 79 patients who underwent curative surgery, 21 (31.8%) had postoperative recurrences. Primary recurrence was found in, lung, liver, lymph node, and local sites for 7, 6, 5, and 5 patients, respectively. The majority of these patients received further treatment but, due the retrospective nature of this study we did not have access to full data regarding post recurrence treatment. As post recurrence therapy could have impacted on overall survival rates., we decided to focus our analysis on relapse free survival (RFS).

In the univariate analysis of RFS, the following parameters were associated with patients’ outcome: pathological stage (and not clinical stage at diagnosis) (hazard ratio (HR) = 2.31, 95% CI:1.02, 5.20, *p* = 0.0435), margins status at the time of resection (HR = 5.63; 95% CI: 1.87,16.96, *p* = 0.0021) and post nCRT neutrophil to lymphocyte ratio (NLR) (HR = 1.04, 95% CI: 1.00, 1.08, *p* = 0.0348).

On the basis of the results obtained in the univariable analyses, pathological stage, margins status and post CRT NLR were included in the multivariable Cox regression model for RFS. Margins status and post nCRT NLR confirmed the statistically significant association with survival. Neither PD-L1 expression nor CD8+ TILs density was significant predictive factors (Table 4).

**Table 4:** Univariate and multivariate analysis of clinicopathologic parameters on RFS

Variable	Univariate			Multivariate		
	HR	95% CI	*p*-Value	HR	95% CI	*p*-Value
All patients (*N* = 79)						
PD-L1 TS (*positive vs. negative*)	1.15	0.53–2.50	0.7238	NA	NA	NA
PD-L1 IF (*positive vs. negative*)	0.92	0.43–1.95	0.8192	NA	NA	NA
CD8-interface (*high vs. low*)	1.00	1.00–1.00	0.1785	NA	NA	NA
Pre CRT NLR (*high vs. low*)	0.80	0.56–1.12	0.1966	NA	NA	NA
Post CRT NLR (*high vs. low*)	**1.04**	**1.00–1.08**	**0.0348**	**1.04**	**1.00–1.09**	**0.0415**
Age *(≥52 vs. <52*)	1.23	0.60–2.51	0.5780	NA	NA	NA
Sex (*male vs female*)	0.57	0.28–1.18	0.1290	NA	NA	NA
Margins (*positive vs negative*)	**5.63**	**1.87–16.96**	**0.0021**	**13.59**	**2.32–79.57**	**0.0038**
Stage at diagnosis (3,4 vs. 1–2)	2.19	0.28–17.35	0.456	1.04	0.00-Inf	1.0000
Interaction between Stage at diagnosis and Radiation	NA	NA	NA	NA	0.85-Inf	1.0000
Pathological stage (*3,4 vs. 0–2*)	**2.31**	**1.02–5.20**	**0.0435**	**1.47**	**0.49–4.40**	0.4862

Abbreviations: CI: confidential interval; CRT: chemoradiation; HR: hazard ratio; IF: interface; NLR: neutrophils to lymphocytes; pStage (pathological stage) TS: tumor stroma.

## DISCUSSION

Within the recent years, immunotherapy with immune checkpoint inhibitors (ICIs) has revolutionized the field of oncology [23–26]. However, despite the current success of immunotherapy, not all patients respond similarly and the benefits of this approach have been limited in non-immunogenic, “cold, tumors [27]. This natural resistance is in part due to various immunosuppressive factors present in the TME that prevent infiltration of CD8+ T cells, unlike in immunogenic tumors. Therefore, to unleash an optimal antitumor immune response, combinatorial approaches that combine immune checkpoints with other modalities, have been investigated and developed [22, 28]. Salient to our work, studies have investigated the immunomodulatory impact of radiation therapy and its ability to alter the immunogenicity of the TME and increase T cell infiltration and antigen processing and presentation [19, 29]. This has paved the way for investigation of combinatorial approaches with immune checkpoint inhibition in different cancer types, and numerous reports have shown clinical benefit of the combination of RT and PD-1/PD-L1 blockade in melanoma [30], non-small cell lung cancer (NSCLC) [31], Hodgkin lymphoma [32], renal cell carcinoma (RCC) [33].

Durable responses, including in non-radiated areas with the use of low dose radiation in combination with ICI therapy, have also been reported in microsatellite stable (MSS) CRC [34].

However, the clinical significance of PD-1(L)1 pathway upregulation in rectal cancers remains controversial and it is important to acknowledge that PD-L1 is an imperfect biomarker and that different analyses have highlighted how immunohistochemistry staining cut off differ among studies and how PD-L1 expression is not uniform, which could induce possible biased results related to sampling [35–38]. However, our findings are consistent with other studies in rectal cancer patients looking at matched pre and post radiation specimens [39–41]. In our analysis, tumor cell PD-L1 expression was overall low (7.7%), which is in line with results presented by other groups [39, 41, 42]. For instance, Hecht et al. reported that the percentage of tumor PD-L1 high expression was 2.1% in rectal cancer [39]. Lee et al. reported that high tumor PD-L1 expression was identified in only 4.8% of the total cohort of rectal cancer [43].

We showed that nCRT exposure was associated with a higher PD-L1 expression in tumor cells as compared to non-nCRT cases. Moreover, although not significant, we observed also an increased overall PD-L1 expression on TS and IF in the nCRT group as compared to patients who had surgery only. CD8+ T cell and their spatial distribution remains a crucial component in eliciting an antitumor immune response [40–42]. Paired analysis of PD-L1 expression and density of CD8+ TILs showed that CRT induce an immunologic shift toward increases in both PD-L1 expression and density of CD8+TILs in rectal cancer patients [43–46]. For this purpose, we developed an IHC approach incorporating both an image analysis approach and a manual approach to quantitate the density of each immune cells’ subsets in different areas of the TME. We applied this approach in this study and were able to assess CD8-TILs in the whole tumor, and in the tumor margins (tumor interface, inner and outer interface). Although no statistically significant differences between the two cohorts were observed, there was a trend toward higher CD8+ cell density in the nCRT cohorts compared to surgery-alone cases, suggesting a different immunological milieu between the two cohorts based on radiation exposure.

Mucins are a class of glycoproteins that play a role in suppressing inflammation abnormal expression of mucins has been observed in various adenocarcinomas, including CRC [47, 48]. Studies have supported mucins’ role in regulating T cells function and modulating immune response, for instance through interaction with intercellular adhesion molecule-1 (ICAM-1) and other inhibitory receptors on T-cells, leading to impaired antigen recognition, [49–51]. Cancer cells can exploit the immune-modulatory ability of mucins to evade immune surveillance [52–55]. We observed a higher mucin concentration in patients who underwent nCRT, as compared to the surgery alone cases. These findings suggest a novel way through which radiation therapy can impact the immune TME towards a more immunosuppressive phenotype.

Finally, our results for both univariate and multivariate analyses indicated that higher post-treatment NLR was an independent predictor of relapse free survival (RFS). Other studies in rectal cancer have focused on pretreatment NLR and showed that elevated higher NLR was associated with higher T stage, inferior RFS, and poorer pathological response to nCRT. These studies have not looked at post treatment NLR. Thus, our findings suggest a possible prognostic role of posttreatment NLR and might help personalizing adjuvant treatments, for instance by intensifying systemic treatment in patients with an elevated NLR after nCRT.

Our study has few limitations that need to be acknowledged. First, we did not have access to pretreatment tumor tissues and the analysis include a limited number of patients, which is a consequence of the retrospective design. It is also important to note that in our study, patients in the nCRT group had a more advanced tumor stage at the time of diagnosis, as compared to the group who received surgery upfront. A possible association between PD-L1 expression and higher TNM stage at diagnosis (regardless of radiation exposure) cannot be excluded, as it has been showed in other tumor types [35–37]. Finally, the variability in test cutoffs and standards for PD-L1 testing should be considered. However, we previously shown that different clones of PD-L1 antibodies, including 5H1, SP142, 28–8, 22C3, and SP263, have similar performance characteristics when used in a standardized IHC assay [38].

In conclusion, our study provides further data on the immunologic impact of nCRT in rectal cancer. We evaluated the effect of nCRT on CD8+TILsPDL-1 expression by spatial localization as well as on mucin expression, and their clinical implications in rectal cancer patients, comparing data from non-CRT cases. Matched pre- and postsurgical specimen analysis with further mechanistic investigations are needed in order to better evaluate the immune milieu of rectal cancer and to expand the applicability of checkpoint inhibitors in this setting.

## MATERIALS AND METHODS

### Patients selection and evaluation

In this retrospective study, we collected surgical specimens from patients with rectal adenocarcinoma treated with surgery with or without nCRT at the Johns Hopkins between 2008 and 2014. We included patients with more evidence of distant metastasis at time of diagnosis, for which we had access to postsurgical tumor tissues obtained and clinicopathologic information. For patient who underwent nCRT further eligibility criteria was the completion of the planned course of preoperative CRT with conventional fractionation plus total mesorectal excision.

Clinical (at the time of diagnosis) and pathologic tumor stages were classified according to the 8th edition of the American Joint Committee on Cancer staging system. Dworak system was used to assess the pathologic regression grade, from 0 (no regression) to 4 (complete pathologic regression). Histological details of the tumors were retrieved from the archived pathological reports. Clinical data were obtained from patient records. The study was approved by the Johns Hopkins Institutional Research Board.

### Immunohistochemical analysis

For immunohistochemistry tissue sections were stained with CD8 and PD-L1 antibodies. Whole slides images were acquired at 20× magnification. The prevalence of positive CD8 stained cells was recorded in the tumor-stroma interface was identified and drew by the pathologist (RAA) based on the H&E staining image and transferred to the analyzed images afterwards in Halo digital analysis software.

To assess if CD8+ cells have been enriched in tissue compartments, we measured the CD8+ cell density in different tissue compartments separately (tumor, interface tumor side, and interface background rectal side); each region was defined as 400 micrometers on both sides of the interface.

The density (# of cells/surface area analyzed) of CD8 expressing lymphocytes was assess via image analysis (HALO Indica Labs). Additionally, before we performed the digital analysis, we first did a quality check by eye with all the images and we didn’t observe a clear cluster pattern of CD8+ cell distribution [56].

PD-L1 membranous expression was manually counted in tumor cells (TC), tumor stroma (TS) and invasive front (IF), as previously described [34, 56]. Two 5 μm-thick sections were cut from one FFPE specimen and mounted on glass slides. After deparaffinization and antigen retrieval, the anti-PD-L1 antibody (SP142, Spring Bioscience) or a concentration matched isotype control were applied and allowed to incubate at 4°C for 22 hours. Signals were developed by using an Avidin Biotin Complex (ABC) Method (Vector Laboratory) combined with the TSA system (PerkinElmer) [56].

The study pathologist (RAA) estimated the percentage of PD-L1 stained cells was estimated on tumor cells (membranous staining), stroma (all the inflammatory, fibroblast and vascular cells, cytoplasm and membranous pattern) and invasive front (stroma between tumor cells and non-tumor tissue). Mucin expression was determined as the percentage of the mucin area in the whole tumor mass area.

### Signal quantitation

Slides were scanned at 20× objective equivalent (0.49 microns/pixel) with Hamamatsu NanoZoomer XR slide scanner. Each image was annotated for regions of tumor, invasive front (400 micron area toward the center of the tumor and 400 micron area outside the tumor edge) and non-tumor regions by the study pathologist (RAA). Positive signals were reported as cell density per mm^2^ tissue area by digital analysis (Halo, Indicalab) [56].

### Statistical analysis

Density and other continuous variables were tested with Student’s *t*-test. Association between categorical variables were analyzed using Fisher exact test with continuity correction. The median value of multiple slides for the same patient were used for analysis.

Pretreatment and post treatment absolute neutrophil count (ANC) absolute and lymphocyte count (ALC) from the peripheral blood were also collected. Pretreatment ANC and ALC values were obtained between the time of cancer diagnosis and treatment initiation (nCRT or surgery). The lab values closest to the time of treatment initiation were used. Post treatment ANC and ALC values were obtained within 60 days from surgery. A pretreatment and post treatment neutrophil-lymphocyte ratio (NLR), NLR was calculated for each patient, defined as the ANC divided by the ALC. NLR was evaluated as a continuous variable. Generalized estimating equations (GEE) were used (assuming a compound symmetry correlation structure) for model estimation and hypothesis testing. Specifically, we modeled the vector of expression from samples among patients as a function of sample location, treatment group, and the interaction of the two. Recurrence free survival (RFS) and overall survival (OS) were analyzed with the Kaplan-Meier method. Univariate and multivariate regression analyses of RFS were performed using Cox’s proportional hazard model. *P*-Values <0.05 were considered to be statistically significant. All the statistical analyses were performed using R 3.5.1.

## Abbreviations

ANC: absolute neutrophil count
ALC: absolute and lymphocyte count
CRC: colorectal cancer
CRT: chemoradiotherapy
CT: chemotherapy
GEE: generalized estimating equations
HPF: high-power fields
HR: hazard ratio
ICI: immune checkpoint inhibitors
IF: invasive front
IFN-γ: interferon-γ
IHC: immunohistochemistry
ICAM: intercellular adhesion molecule-1
LARC: locally advanced rectal cancer
MSI: microsatellite instable
MSS: microsatellite stable
N: lymph node
nCRT: neoadjuvant chemoradiotherapy
NLR: neutrophil to lymphocytes ratio
NSCLC: non-small cell lung cancer
OS: overall survival
PD-1: programmed death 1
PD-L1: programmed death ligand-1
RCC: renal cell carcinoma
RFS: recurrence-free survival
RT: radiation therapy
TC: tumor cells
TILs: tumor-infiltrating lymphocytes
TME: tumor microenvironment
TRG: tumor regression grade
TS: tumor stroma
